# Overexpression of Periostin and Lumican in Esophageal Squamous Cell Carcinoma

**DOI:** 10.3390/cancers2010133

**Published:** 2010-03-01

**Authors:** Manoj Kumar Kashyap, Arivusudar Marimuthu, Suraj Peri, Ghantasala S. Sameer Kumar, Harrys K.C. Jacob, Thottethodi Subrahmanya Keshava Prasad, Riaz Mahmood, K. V. Veerendra Kumar, M. Vijaya Kumar, Stephen J. Meltzer, Elizabeth A. Montgomery, Rekha V. Kumar, Akhilesh Pandey

**Affiliations:** 1 Institute of Bioinformatics, International Technology Park, Bangalore 560066, India; E-Mails: manoj@ibioinformatics.org (M.K.K.); arivusudar@ibioinformatics.org (A.M.); sameer@ibioinformatics.org (G.S.S.K.); keshav@ibioinformatics.org (T.S.K.P.); 2 McKusick-Nathans Institute of Genetic Medicine, Johns Hopkins University School of Medicine, Baltimore, MD 21205, USA; E-Mail: harrys@jhmi.edu (H.K.C.J.); 3 Department of Biological Chemistry, Johns Hopkins University School of Medicine, Baltimore, MD 21205, USA; 4 Department of Oncology, Johns Hopkins University School of Medicine, Baltimore, MD 21205, USA; E-Mail: smeltzer@jhmi.edu (S.J.M.); 5 Department of Medicine, Johns Hopkins University School of Medicine, Baltimore, MD 21205, USA; 6 Department of Pathology, Johns Hopkins University School of Medicine, Baltimore, MD 21205, USA; E-Mail: emontgom@jhmi.edu (E.A.M.); 7 Department of Biotechnology, Kuvempu University, Shimoga District, Karnataka 577451, India; E-Mail: riaz_sultan@yahoo.com (R.M.); 8 Biostatistics and Bioinformatics, Fox Chase Cancer Center, Philadelphia, PA 19111-2497, USA; E-Mail: Suraj.Peri@fccc.edu (S.P.); 9 Department of Surgical Oncology, Kidwai Memorial Institute of Oncology, Bangalore, Karnataka 560029, India; E-Mails: kidwai@kar.nic.in (K.V.V.K.); vijai2002@yahoo.com (M.V.); 10 Department of Pathology, Kidwai Memorial Institute of Oncology, Bangalore, Karnataka 560029, India

**Keywords:** stroma, extracellular matrix, DNA microarrays, epithelial-mesenchymal transition

## Abstract

To identify biomarkers for early detection for esophageal squamous cell carcinoma (ESCC), we previously carried out a genome-wide gene expression profiling study using an oligonucleotide microarray platform. This analysis led to identification of several transcripts that were significantly upregulated in ESCC compared to the adjacent normal epithelium. In the current study, we performed immunohistochemical analyses of protein products for two candidates genes identified from the DNA microarray analysis, periostin (*POSTN*) and lumican (*LUM*), using tissue microarrays. Increased expression of both periostin and lumican was observed in 100% of 137 different ESCC samples arrayed on tissue microarrays. Increased expression of periostin and lumican was observed in carcinoma as well as in stromal cell in the large majority of cases. These findings suggest that these candidates can be investigated in the sera of ESCC patients using ELISA or multiple reaction monitoring (MRM) type assays to further explore their utility as biomarkers.

## 1. Introduction

ESCC is the predominant type of esophageal cancer in the developing world [1]. We previously reported identification of a number of genes significantly upregulated in ESCC using DNA microarrays [2]. Among these were several differentially expressed ECM genes such as matrix metalloproteinase 13 (*MMP13*), hyaluronic acid synthase 3 (*HAS3*), aggrecan 1 chondroitin sulfate proteoglycan 1 (*AGC1*), secreted protein acidic cysteine rich osteonectin (*SPARC*), matrix metalloproteinase 11 (*MMP11*), serine or cysteine proteinase inhibitor clade b ovalbumin member 11 (*SERPINB11*) and epithelial membrane protein 1 (*EMP1*) [2].

Components of the extracellular matrix (ECM) are key molecules in tumor-stroma interactions, which are now believed to be involved in progression of different tumor types. Thus, we chose to validate two ECM molecules that were highly expressed in almost all ESCC samples at the mRNA level. Periostin is a secreted extracellular matrix protein that belongs to the fasciclin gene family. Periostin is a disulfide linked 90 kDa proteins that functions as a cell adhesion molecule. Lumican belongs to a family of small leucine-rich repeat proteoglycans that includes keratocan, mimecan, decorin, fibromodulin, biglycan, and proline arginine rich end leucine-rich protein. We observed periostin and lumican to be expressed at high levels in 137 out of 137 ESCC tumors tested.

## 2. Results

**Figure 1:**
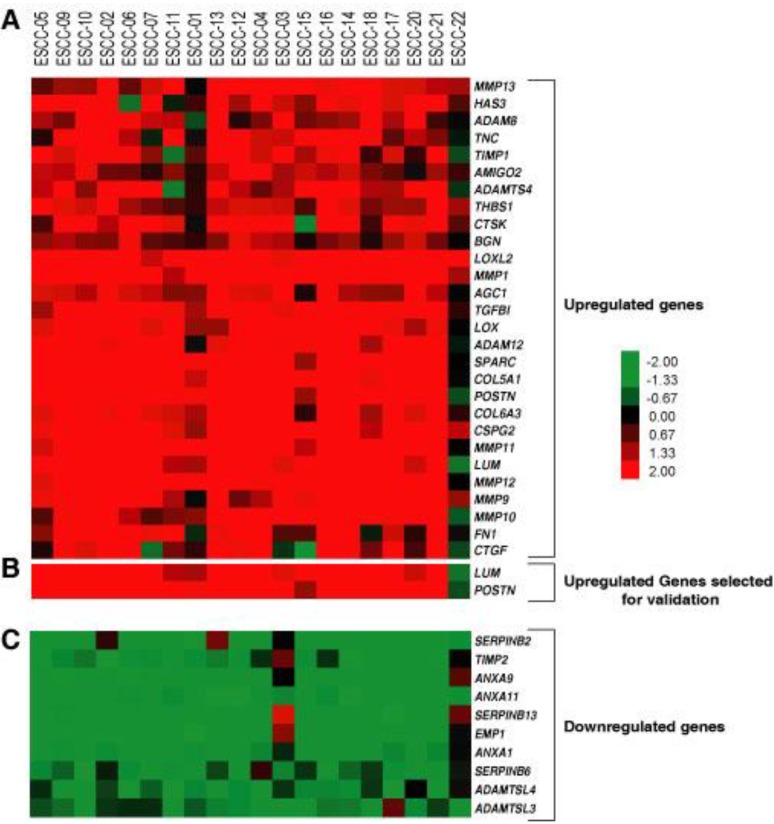
Hierarchical clustering of extracellular matrix-related genes in esophageal squamous cell carcinoma. **(A)** *Panel A* shows upregulation of extracellular matrix-related genes in 20 patients with esophageal squamous cell carcinoma. **(B)** *Panel B* shows mRNA expression levels of *POSTN* and *LUM* genes. The upregulated genes were represented with a p-value < 0.001 and a fold-change ≥2. **(C)** *Panel C* shows mRNA expression levels of downregulated genes in ESCC with a p-value < 0.001 and a fold-change ≤0.5.

Using whole human genome oligonucleotide microarrays, we have previously reported gene expression profiling of 20 ESCC cases along with their adjacent normal epithelia as controls. We chose to evaluate extracellular matrix related genes that were overexpressed in ESCC as candidate biomarkers. Figure 1A shows a heatmap for upregulated extracellular matrix related genes from this analysis. The mRNA expression profiles of periostin and lumican across the samples are shown in Figure 1B. On average, *POSTN* was upregulated 11-fold whereas *LUM* was upregulated 7-fold in ESCC as compared to the adjacent normal epithelia. Figure 1C shows a heatmap for downregulated extracellular matrix related genes from this analysis.

To clinically validate these findings from DNA microarrays in a larger cohort, we used commercially available tissue microarrays (TMAs) to test the pattern of expression of periostin and lumican.

### 2.1. Validation of Periostin Overexpression in ESCC

**Figure 2:**
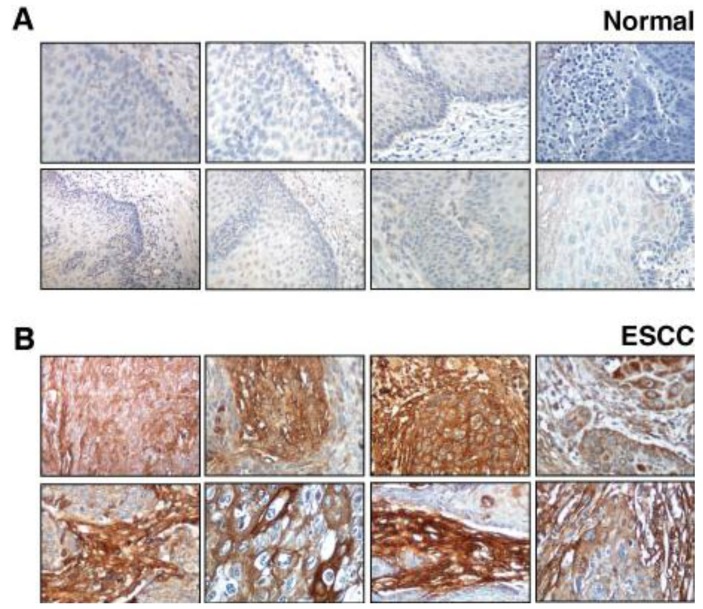
Periostin expression in a group of normal esophagus and ESCC. **(A)** *Panel A* shows expression of periostin in representative normal esophageal squamous mucosa. **(B)** *Panel B* shows expression of periostin in stromal and epithelial cell compartment of ESCC cases. The staining pattern was cytoplasmic and/or membranous.

We performed immunohistochemical labeling of tissue microarrays using commercially available antibodies against periostin and lumican. We observed overexpression of periostin in both tumor and stromal cells. The staining pattern of periostin in a few representative sections of normal esophagus and ESCC is shown in Figure 2. A total of 137 ESCC sections (all from different patients) were stained and analyzed. High expression of periostin was observed in 100% of ESCC cases. In 72% of ESCC cases, expression of periostin was detected both in tumor and stromal cells. In 11% of ESCC cases, the staining was restricted to carcinoma cells, while in 17% of ESCC cases it was observed only in the stroma. Expression of periostin was detected in 6% (7/112) of normal esophageal epithelia. 

### 2.2. Validation of Lumican Overexpression in ESCC

**Figure 3:**
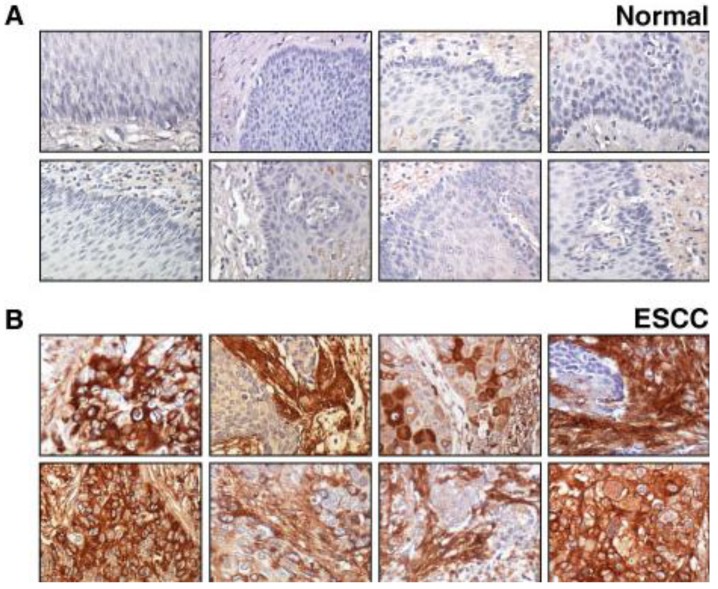
Lumican expression in a group of normal esophagus and ESCC. **(A)** *Panel A* shows expression of lumican in normal esophageal squamous mucosa. **(B)** *Panel B* shows expression of lumican in stromal and epithelial compartment of ESCC cases. The staining pattern was cytoplasmic and/or membranous.

We again used the tissue microarray platform to validate lumican expression as a potential biomarker for ESCC. Figure 3 shows the staining pattern of lumican in several representative sections of normal epithelia and carcinomas. Expression of lumican was observed in all 137 ESCC cases. In 69% of ESCC cases, expression of lumican was found to be high in carcinoma as well as in carcinoma-associated stromal cells. In 14% of ESCC cases, the high expression of lumican was restricted to carcinoma cells while in 17% of ESCC cases; the increased expression was restricted to stromal cells. Expression of lumican was observed in 7% (8/112) of normal esophageal epithelia. In parallel, we used two types of negative controls, matched isotype antisera instead of the primary antibodies or diluent instead of the primary antibodies, and did not observe any appreciable staining in the cancer specimens confirming the specificity of our staining. IHC labeling data for both the molecules is summarized in Table 1.

**Table 1:** Expression pattern of periostin and lumican among ESCC cases.

	Parameters	Periostin	Lumican
**1**	Number of cases tested	137	137
**2**	Number of positive cases	137	137
**3**	Number of cases with positive staining of carcinoma cells alone	15	19
**4**	Number of cases with positive staining of stroma alone	23	24
**5**	Number of cases with positive staining of both carcinoma and stromal compartments	99	94

**Figure 4:**
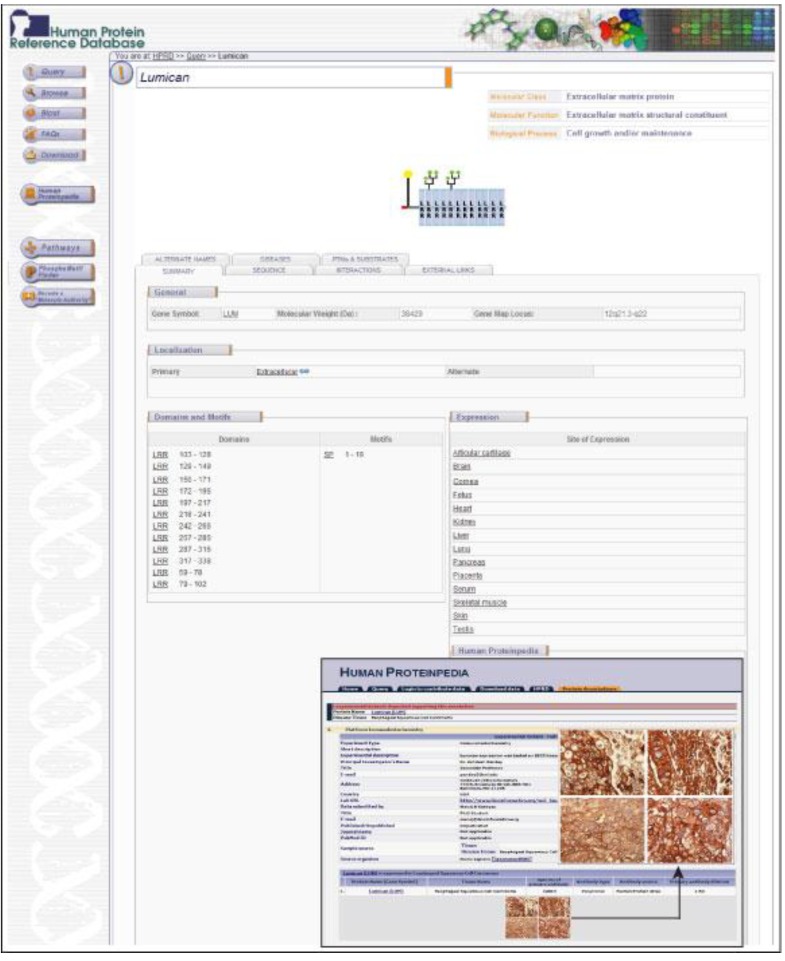
A snapshot of lumican annotation in human Proteinpedia. Shown here is the data for lumican from human Proteinpedia with permission, a public portal that provides data for a given protein from different experimental platforms. The figure shows the immunohistochemical staining of lumican in selected esophageal squamous cell carcinoma tissues.

To make our observation publicly available and accessible to other researchers, we have submitted our data on immunohistochemical analysis of periostin and lumican to Human Proteinpedia (www.humanproteinpedia.org) [3]. Figure 4 shows a screenshot of this resource displaying lumican expression in ESCC.

## 3. Material and Methods

### 3.1. DNA Microarray Analysis

DNA microarray data was processed and analyzed using the R programming platform and Bioconductor packages. The differentially expressed extracellular matrix related genes were identified between ESCC and matched normal samples using Bayes moderated t-test as described previously [2]. The differentially expressed genes in ECM of ESCC versus adjacent normal were filtered based on a p-value threshold of 0.001 and a fold-change cutoff of ≥2 or ≤0.5 for upregulated and downregulated genes, respectively.

### 3.2. Tissue Samples

This study was approved by the Institutional Review Board of the Kidwai Memorial Institute of Oncology, Bangalore and the Johns Hopkins School of Medicine, Baltimore. Commercially available tissue microarrays were obtained from CreativeTM Biolabs (catalog no. CBL-TMA-046), consisting of 64 ESCCs, 3 esophageal adenocarcinomas, and 3 normal esophageal tissues. All samples were fixed by overnight incubation in 10% buffered formalin. The ESCCs were well to poorly differentiated from patients aged 41 to 75 years. A second set of TMAs were obtained from Pantomics (catalog no. ESC96101), which consisted of 34 cases in duplicate, ranging from differentiation grades I to III from patients ages 36 to 71 years. A third set of tissue microarrays were obtained from FolioBio (catalog no. ARY-HH0091), which consisted of 40 ESCCs with matched adjacent normal esophageal squamous epithelium. All patients were stage T3N1M0 and with histopathological grades ranging from well differentiated to poorly differentiated.

### 3.3. Immunohistochemical Staining

Immunohistochemical staining was performed on paraffin-embedded sections for periostin and lumican. A rabbit polyclonal antibody against periostin was purchased from Biovendor Laboratory (catalog # RD181045050) and used at a dilution of 1:500. A rabbit polyclonal antibody against lumican was purchased from the Human Protein Atlas (catalog # HPA001522b) and used at a dilution of 1:50. An Envision kit (DAKO) was used according to the manufacturer’s instructions. Briefly, formalin fixed paraffin-embedded tissue sections were deparaffinized and antigen retrieval was carried out by incubating for 20 minutes in antigen retrieval buffer. Endogenous peroxidases were quenched using the blocking solution followed by three washes with the wash buffer. The sections were incubated overnight at 4^o^C in a humidified chamber with the primary antibodies. After washing, the slides were incubated with appropriate horseradish peroxidase conjugated secondary antibody for 30 minutes at room temperature. Immunoperoxidase staining was developed for 5 minutes using DAB chromogen, and counterstained with hematoxylin. Immunohistochemical labeling was assessed by an experienced expert pathologist (RVK), and intensity of staining was scored as negative (0), mild (1+), moderate (2+), or strong (3+). The distribution of staining of cancer cells was scored as 0 (less than 5% of cells staining), 1+ (5%–30% of cells staining), 2+ (31%–60% of cells staining) or 3+ (greater than 60% of cells staining). Comparisons were made between the intensity of the staining of carcinoma cells and that of normal esophageal epithelium. The criterion for scoring the stroma was also the same as for the carcinoma cells. We used two types of negative controls—matched isotype antisera instead of the primary antibodies or diluent instead of the primary antibodies.

## 4. Conclusions

Extracellular matrix proteins are increasingly being demonstrated to be involved in the process of epithelial-to-mesenchymal transition events leading to tumor progression. ECM proteins have been suggested as attractive targets as biomarkers because they are more easily detected in body fluids such as serum as compared to intracellular proteins. Here we chose *POSTN* and *LUM* genes, which were identified from a previous microarray study, for further validation using a tissue microarray platform.

Periostin is a secreted mesenchyme-specific protein involved in regulation, adhesion and differentiation of osteoblasts and wound repair [4,5]. It has been shown to be involved in epithelial-to-mesenchymal transition in cancers [6] and to be involved in tumor angiogenesis [7]. Experimental evidence suggests that periostin promotes cell migration, motility, adhesion and metastatic cell growth of the tumors [8,9]. Eosinophilic esophagitis is an inflammatory disease, which is sometimes misdiagnosed as gastroesophageal reflux diseases (GERD) [11,12]. Overexpression of both periostinmRNA and protein expression levels in eosinophilic esophagitis as compared to the normal esophageal epithelium has been described previously [11,13]. In a study on Barrett’s esophagus, a 7-fold transcriptional upregulation of *POSTN* was reported as compared to normal esophageal tissue [10]. In our gene expression analysis, *POSTN* was found to be 11-fold upregulated in ESCC [2]. Elevated levels of serum *POSTN* have been reported in breast cancer patients presenting with bone metastases suggesting that *POSTN* could also be further investigated as a potential metastatic biomarker in the sera of ESCC patients [14].

Lumican belongs to the same molecular class as periostin and is also a secreted protein. Gene expression profiling studies of Barrett’s esophagus and esophageal adenocarcinoma have reported a transcriptional upregulation (~5-fold) of *LUM* in tumors as compared to adjacent normal esophageal epithelium [10]. In our previous study on gene expression analysis of ESCC, we also observed a 7-fold upregulation of *LUM*. Based on the above findings, we hypothesize that lumican could also be a potential biomarker if it could be detected in ESCC patient’s sera.

The functional significance of these targets in ESCC tumorigenesis requires further investigation to elucidate their roles in tumor progression and metastasis. Nevertheless, our findings support the rationale for further evaluation of periostin and lumican in the sera of ESCC patients for diagnostic purposes.
